# Comparative Dynamic Pulmonary Function Tests Between Apparently Healthy Young Adult Offspring of Asthmatic and Non-asthmatic Parents: A Pilot Study

**DOI:** 10.7759/cureus.44259

**Published:** 2023-08-28

**Authors:** Reddipogu Havilah Twinkle, Yukta Sain, Mohammed Jaffer Pinjar, Khaleel Ahmed Manik, Himel Mondal

**Affiliations:** 1 Physiology, Great Eastern Medical School and Hospital, Ragolu, IND; 2 Medical School, Great Eastern Medical School and Hospital, Ragolu, IND; 3 Physiology, All India Institute of Medical Sciences, Deoghar, Deoghar, IND; 4 Physiology, Integral Institute of Medical Sciences & Research, Faculty of Medicine & Health Sciences, Integral University, Lucknow, IND

**Keywords:** allergy, genetic factors, young adult, obstruction, asthma, lung, respiratory function tests, vital capacity, exhalation, forced expiratory volume

## Abstract

Background

While the hereditary component of asthma has been established, its influence on early respiratory function changes in otherwise healthy offspring remains to be explored. Dynamic lung function tests assess airflow in and out of the lungs, providing valuable insights into respiratory health and detecting potential airflow limitations. This study aimed to compare the dynamic lung functions between offspring of asthmatic and non-asthmatic parents.

Methodology

A case-control design was employed comprising 30 cases (offspring of asthmatic parents) and 30 controls (offspring of non-asthmatic parents). Lung function parameters including forced vital capacity (FVC), forced expiratory volume in one second (FEV1), FEV1/FVC ratio, forced expiratory flow between 25% and 75% of the FVC (FEF 25-75%), and maximum mid-expiratory flow at 50% of the FVC (Vmax 50%) were measured. Statistical analysis was conducted to compare the parameters between cases and controls using the unpaired t-test.

Results

The mean age of controls was 20.46 ± 2.82 years and the cases was 19.83 ± 1.41 years. The study revealed that cases exhibited lower FEV1 and Vmax 50% values compared to controls, indicating potential airflow limitations and altered mid-exhalation flow rates in the offspring of asthmatic parents. While trends were observed in FVC, FEV1/FVC ratio, and FEF 25-75%, these differences were not statistically significant.

Conclusions

The findings suggest a potential association between parental asthma and altered lung function parameters, specifically in FEV1 and Vmax 50%, among their offspring. These early respiratory function changes underscore the potential impact of hereditary factors on lung health. Healthcare professionals should take parental asthma into account when evaluating lung functions. This may lead to earlier detection and intervention. Further investigation is warranted to elucidate the underlying mechanisms and long-term implications of these findings.

## Introduction

Respiratory diseases, particularly asthma, constitute a significant global health concern, affecting millions of individuals of all ages [1]. The intricate interplay between genetic and environmental factors in asthma development has long intrigued researchers seeking to unravel the intricate web of causation [2]. Genetic susceptibility, often attributed to a combination of multiple genes, has been identified as a significant risk factor in asthma inheritance. With its complex etiology and multifaceted pathophysiology, asthma has been the subject of extensive research aimed at understanding its underlying mechanisms and exploring potential risk factors [3].

Dynamic lung function tests, also known as pulmonary function tests, offer a range of advantages that are crucial for assessing respiratory health and diagnosing various lung conditions [4]. These tests provide valuable insights into how efficiently an individual’s lungs are functioning, allowing healthcare professionals to evaluate lung capacity, airflow rates, and the overall mechanics of breathing. Moreover, dynamic lung function tests are instrumental in differentiating between obstructive and restrictive lung diseases, aiding in accurate diagnosis and tailored treatment plans [5]. Their quantitative nature ensures objective measurements, facilitating the tracking of treatment effectiveness over time.

Offspring of asthmatic parents are at an increased risk of inheriting a genetic predisposition to asthma. As a result, these individuals might exhibit reduced pulmonary function, characterized by decreased lung capacity and potential airway hyperresponsiveness [6]. This may lead to a higher likelihood of developing asthma or related respiratory conditions later in life.

By comparing the pulmonary function tests of healthy offspring born to parents with asthma and those without asthma, this study seeks to identify any early signs of lung function abnormalities or differences that might be linked to genetic predisposition. This research holds important implications for understanding the inheritability of lung function traits and the potential risk factors associated with asthma development. If the study reveals significant differences between these two groups, it could provide valuable insights into the underlying mechanisms of asthma and aid in identifying individuals at higher risk, enabling early interventions and preventive measures. Ultimately, the findings from this pilot study could contribute to more personalized healthcare approaches for individuals with a family history of asthma and potentially lead to advancements in asthma prevention and management strategies.

## Materials and methods

Study type and settings

This was a cross-sectional study with a group of research participants as cases and another age-matched group as controls. The study was performed from November to December 2022. The study was conducted at Great Eastern Medical School and Hospital, Srikakulam, Andhra Pradesh, India.

Study sample

The study population consisted of healthy individuals aged 18 to 25 years. The cases comprised 30 healthy offspring of parents (any parent with a history of asthma diagnosed before the birth of the child) with asthma, while the controls included 30 healthy individuals matched for age and gender whose parents did not have asthma or other non-communicable diseases.

A hospital-based convenience sample was taken from the patient's attendants. Any healthy individuals with or without a history of asthma among their parents who voluntarily provided informed consent and whose age was between 18-25 years were included in the study. Anyone who was a smoker, alcoholic, had undergone surgery, had congenital disorders, had experienced respiratory infections such as tuberculosis, or displayed symptoms of pain, nausea, or altered mental status was excluded from the study.

Dynamic lung function tests

Tests were performed to record pulmonary function parameters, including forced expiratory volume in one second (FEV1), forced vital capacity (FVC), FEV1/FVC ratio, forced expiratory flow between 25% and 75% of the FVC (FEF 25-75%), and maximum mid-expiratory flow at 50% of the FVC (Vmax 50%). We used Clarity SpiroTech Model number CMSP-02 (Punjab, India) which uses flow turbine sensors for measuring the flow rate.

Data analysis

Data were expressed as mean and standard deviation. An unpaired t-test was used to compare the parameters between cases and controls. We used GraphPad Prism 9.5.0 for conducting statistical tests. A p-value <0.05 was considered statistically significant.

Ethics

The study’s purpose was communicated to participants and their informed consent was obtained. Ethical clearance was obtained from the Institutional Ethical Committee of Great Eastern Medical School and Hospital (approval number: 119/IEC/GEMS&H/2022 issued on 09/11/2022).

## Results

A total of 15 males and 15 females comprised the control group, and their mean age was 20.46 ± 2.82 years. A total of 13 males and 17 females comprised the case group, and their mean age was 19.83 ± 1.41 years. The age, height, weight, and body mass index (BMI) are shown in Table 1.

**Table 1:** Age and anthropometric parameters in controls and cases. P-values are calculated from the unpaired t-test.

Parameter	Control (n = 30)	Case (n = 30)	P-value
Age (years)	20.46 ± 2.82	19.83 ± 1.41	0.1
Height (cm)	161.56 ± 21.21	160 ± 12.02	0.54
Weight (kg)	63.9 ± 0	59.68 ± 20.15	0.19
Body mass index (kg/m^2^)	24.8 ± 8.62	23.28 ± 4.45	0.29

There were variations in pulmonary function parameters between healthy offspring of asthmatic parents and those without a family history of asthma, as shown in Table 2.

**Table 2:** Pulmonary function test parameters in controls and cases. FEV1: forced expiratory volume in one second; FVC: forced vital capacity; FEV1/FVC: the ratio of forced expiratory volume in one second to forced vital capacity; FEF 25-75%: forced expiratory flow between 25% and 75% of the FVC; Vmax 50%: maximum mid-expiratory flow at 50% of the FVC

Parameter	Control (n = 30)	Case (n = 30)	Cohen’s d	P-value
FVC (L)	2.81 ± 0.08	2.53 ± 0.46	0.85	0.08
FEV1 (L)	2.48 ± 0.22	2.2 ± 0.42	0.84	0.049*
FEV1/FVC	89.09 ± 4.49	87.09 ± 1.54	0.6	0.36
FEF 25–75% (L/s)	3.35 ± 0.80	2.83 ± 1.80	0.37	0.058
Vmax 50% (L/s)	3.64 ± 0.13	2.96 ± 0.84	1.13	0.019*

While the FVC and FEV1/FVC ratio did not show statistically significant differences, the FEV1 displayed a significant reduction in healthy offspring with asthmatic parents compared to controls. Moreover, the Vmax 50% was notably lower in the asthmatic offspring group, signifying potential respiratory flow limitations.

## Discussion

The significant finding of reduced FEV1 among healthy offspring of asthmatic parents compared to controls could be attributed to a combination of genetic predisposition and potential early respiratory effects. Genetic factors inherited from asthmatic parents may contribute to alterations in lung structure or function, potentially leading to reduced FEV1 values in the offspring [7]. Additionally, exposure to environmental factors or subclinical inflammation due to genetic susceptibility could play a role [8]. The decreased Vmax 50% in the asthmatic offspring group might indicate early airflow limitation, which could be influenced by inherited traits affecting airway resistance. While these findings are suggestive of hereditary influences on lung function, further research is needed to unravel the intricate interplay between genetic and environmental factors that contribute to these differences as well as to ascertain whether they are predictive of future respiratory health issues [9].

Reduced FEV1 and Vmax 50% typically point to the presence of obstructive lung diseases such as asthma and chronic obstructive pulmonary disease. These conditions are characterized by airflow limitations caused by airway inflammation, bronchospasms, and structural changes in the lungs. In asthma, for instance, narrowed airways and increased mucus production lead to decreased FEV1 as well as diminished Vmax 50%, indicating difficulty in exhaling air efficiently. These pulmonary function changes serve as crucial indicators of respiratory dysfunction, warranting comprehensive medical assessment to determine the exact diagnosis and appropriate management strategies [10,11].

Several studies provide insights into the diverse factors influencing asthma severity and development. Genetics influence overall asthma symptom severity variation, including wheezing and shortness of breath [12]. Obesity from childhood to adolescence predicts increased asthma incidence, potentially due to elevated proinflammatory serum leptin levels [13,14]. Maternal smoking during pregnancy is associated with adverse fetal outcomes and heightened childhood wheezing and asthma risk, independent of birth weight and pregnancy duration [15]. Occupational exposure contributes to adult-onset asthma, and developing countries like India have a high level of air pollution that may contribute to the development of adult-onset asthma [16].

When parents have asthma but their babies appear to be normal, it is wise to take certain precautions to ensure a healthy environment. Prioritize a smoke-free home to prevent exposure to harmful tobacco smoke and maintain good indoor air quality by using air purifiers and avoiding strong odors. Control allergens through regular cleaning, washing bedding in hot water, and ventilation. Keep up with vaccinations, including flu shots, to safeguard against respiratory infections [17,18]. Encourage a healthy lifestyle for both parents and babies with balanced diets and regular exercise. Create an asthma action plan for parents, ensuring caregivers are familiar with it. Maintain open communication with babies as they grow, educate them about asthma, and promote stress management techniques [19]. While there is no need for undue worry, these precautions can contribute to a safe and supportive environment for the respiratory well-being of the babies. If any respiratory issues arise, consulting a pediatrician for guidance is advisable [20].

Several limitations should be acknowledged in this study. The sample size, while appropriate for the study’s scope, might limit the detection of nuanced effects and the generalizability of findings to larger populations. Sampling bias could arise if participants do not accurately represent the broader population under investigation. Additionally, potential measurement errors in data collection methods and instruments could introduce inaccuracies in the results. The study’s cross-sectional design may restrict its ability to establish causal relationships or capture changes over time. Moreover, the reliance on participants’ recall might introduce recall bias, impacting the accuracy of reported information. Ethical concerns and time constraints could also influence the study’s outcomes. Recognizing and addressing these limitations is crucial for a comprehensive and accurate interpretation of the study’s findings.

## Conclusions

The study revealed that the offspring of asthmatic parents displayed lower FEV1 and Vmax 50% values compared to controls. These reductions indicate potential airflow limitations and altered mid-exhalation flow rates. While trends were observed in other parameters, these findings underscore the potential impact of parental asthma on early respiratory function changes in their offspring. Healthcare professionals should take parental asthma into account when evaluating lung functions. This may lead to earlier detection and intervention enabling tailored preventive measures and treatments. Furthermore, these findings emphasize the need for ongoing research to develop a deeper understanding of the mechanisms involved in hereditary predisposition to altered lung function.
